# Phytochemical characterization of oil and protein fractions isolated from Japanese quince (*Chaenomeles japonica*) wine by-product

**DOI:** 10.1016/j.lwt.2023.114632

**Published:** 2023-03-15

**Authors:** Sana Ben-Othman, Uko Bleive, Hedi Kaldmäe, Alar Aluvee, Reelika Rätsep, Kadri Karp, Larissa Silva Maciel, Koit Herodes, Toonika Rinken

**Affiliations:** a ERA Chair for Food (By-) Products Valorisation Technologies, Estonian University of Life Sciences, Kreutzwaldi 56/5, 51006, Tartu, Estonia; b Polli Horticultural Research Centre, Chair of Horticulture, Institute of Agricultural and Environmental Sciences, Estonian University of Life Sciences, Uus 2, 69108, Polli, Estonia; c Chair of Horticulture, Institute of Agricultural and Environmental Sciences, Estonian University of Life Sciences, Kreutzwaldi 5, Tartu, 51006, Estonia; d Institute of Chemistry, University of Tartu, Ravila 14a, 50411, Tartu, Estonia

**Keywords:** Japanese quince, By-product valorisation, Supercritical CO_2_ extraction, Oil fraction, Protein isolate

## Abstract

The wine industry generates large quantities of by-products each year. Therefore, this work aimed to isolate and evaluate the oil and protein fractions of Japanese quince (*Chaenomeles japonica*, JQ) press residue, offering a partial utilization of valuable bioactive compounds of wine industry by-products. To study the JQ oil extract yield, composition and oxidation stability, we modified the co-solvent composition during the supercritical CO_2_ (SC–CO_2_) extraction of oil by adding different ethanol content. The remaining defatted material was used for the isolation of proteins. The SC-CO_2_ extraction yielded oil rich in polyunsaturated fatty acids, tocopherols, and phytosterols. The use of ethanol as a co-solvent increased the oil yield but did not enhance its oxidative stability or content of antioxidants. We recovered protein isolate after removing tannins with 70% ethanol extraction in the next step. The JQ protein isolate contained all essential amino acids. In addition to its balanced amino acid composition, the protein isolate exhibited excellent emulsifying properties highlighting its potential as a food additive. In conclusion, JQ wine by-products can be utilized for the extraction of oil and protein fractions which can be used in food or cosmetic product formulation.

## Highlights

• Consecutive extraction of oil and protein from Japanese quince pressing residue.
• Ethanol as SC-CO_2_ extraction co-solvent increased oil yield, but not its stability.
• The oil is rich in polyunsaturated fatty acids, tocopherols, and phytosterols.
• Protein isolate containing all essential amino acids was an excellent emulsifier.
• Oil and protein fractions did not contain the cyanogenic glycoside amygdalin.

## 1 Introduction

The production of wines from local fruits and berries has become popular during the last decade in Estonia due to the raw material's high quality and unique composition. Japanese quince (*Chaenomeles japonica* (Thunb.) Lindl.; JQ) is famous for its original and intensive taste making it a well-appreciated material in wine production. The small apple-shaped fruits of JQ weigh below 50 g and are about 4 cm in diameter (Rumpunen, 2002). The fruit is from yellow-green to dark yellow with some citrus-like aroma. *Chaenomeles* are native to temperate areas, spreading mainly over China, Korea and Japan (Miao, Zhao, Gao, Wang, & Gao, 2018). The main areas for JQ cultivation in Europe are the northern countries, such as Finland, Sweden, Estonia, Latvia, Lithuania, and Poland (Rumpunen, 2002). Many cultivars are already available for cultivation in cool climate conditions, such as ‘Rasa’, ‘Cido’, ‘Cido Red’, and ‘Rondo’. JQ has the nickname “Nordic lemon” due to its sour taste and strong fruit flavour. In Estonia, JQ is in sixth place as a raw material for fruit wine production after apple, rhubarb, black currant, chokeberry and raspberry, with 2200 L in 2020 (Eesti Veinitee). The exact number of plantations and hectares under cultivation is difficult to estimate because factual data and statistics on JQ are unavailable. The fruits of JQ have been used for different products, starting from traditional juices and syrups, while candied fruit snacks and chips have been gaining popularity in recent years.

Different parts of JQ fruit are used to extract value-added bioactive compounds. The fruit peel and pulp have been used for the extraction of polyphenols mainly consisting of phenolic acids (chlorogenic acid, p-coumaric acid, syringic acid) and polymeric proanthocyanidins, also known as condensed tannins (Byczkiewicz, Szwajgier, Kobus-Cisowska, Szczepaniak, & Szulc, 2021; Turkiewicz, Wojdyło, Tkacz, & Nowicka, 2021). Such extracts are reported for their antioxidant and antimicrobial activities (Urbanavičiūte et al., 2020). JQ fruit extracts also contain triterpenes (oleanolic and ursolic acid), known for their anti-inflammatory and anti-hyperlipidemic effects (Du et al., 2013). Moreover, JQ fruit is a good source of pectin, containing up to 1.4 g pectins/100 g fresh fruit (Thomas, Guillemin, Guillon, & Thibault., 2003).

JQ seeds, constituting up to 9% of fresh fruit weight, are used to extractoil, rich in polyunsaturated fatty acids and lipophilic bioactive compounds like tocopherols, phytosterols, and carotenoids (Górnaś, Siger, & Segliņa, 2013). Compared to other fruit seed oils (sea buckthorn, grape, gooseberry and apple), JQ oil shows the highest concentration of monounsaturated fatty acids (Górnaś & Rudzińska, 2016). According to Górnaś et al. (2014), JQ seed oil contains 13 fatty acids, of which palmitic acid (10.07%), oleic acid (34.55%), and linoleic acid (52.35%) are predominating. JQ seed oil is also rich in minor lipophilic compounds like α-tocopherol and β-sitosterol (Mišina et al., 2021). The JQ oil content is related to the abiotic factors affecting the fruits during ripening and harvesting, and the differences in oil levels have been 10–50% (Sipeniece et al., 2021).

Different extraction techniques have been evaluated for the recovery of oil from seeds; Soxhlet extraction with organic solvents gave the highest yield (11.5%), followed by SC-CO_2_ extraction (9.4%), while cold press extraction had the lowest yield (8.7%) (Górnaś et al., 2019). In addition to lipid fraction, JQ seeds are reported to be a good source of polyphenols (up to 2.45 mg/g seeds), proteins (containing all essential amino acids), microelements (Fe, Cu, Zn, and Mn), and polysaccharides (seed mucilage) (Turkiewicz et al., 2021; Ubranavičiūtė & Viškelis, 2022). However, when utilising JQ seeds, caution should be taken due to the presence of cyanogenic glycosides (mainly amygdalin) that can be metabolized in the human digestive system to poisonous hydrogen cyanide (HCN) (Turkiewicz et al., 2021). JQ seeds contain up to 254 mg HCN/kg, which remain in the defatted meal after oil extraction (Mierina et al., 2013).

In the present study, we evaluated the potential utilization of the JQ pressing residue of cultivar ‘Rasa’, containing seeds, peel, and pulp, for the multistep extraction of oil and protein fractions. For the initial step - supercritical extraction, different extraction conditions in terms of different co-solvent contents were used for the recovery of oil fraction. The remaining defatted JQ press residue underwent ultrasound-assisted extraction to remove the tannins and cyanogenic glycosides, followed by the aqueous extraction of proteins.

## 2 Materials and methods

### 2.1 Material

JQ fruits of the ‘Rasa’ cultivar were harvested in south Estonia (Kanepi Parish, Estonia, 58°01′N 26°40′E) on 25.09.2021. The JQ residue was obtained after fruit wine production. The fresh fruits were smashed using a centrifugal mill (RM1.5, Voran Maschinen GMBH, Austria) and treated for 7 d with EnartisZym 1000S (2 g enzyme per 100 kg smashed fruits) at 18–20 °C. The residue obtained after juice pressing (16% of the initial fruit mass) was air-dried in a lab drier for 50 h at 26 °C, vacuum-packed and stored at 4 °C until extraction.

**Fig. 1:**
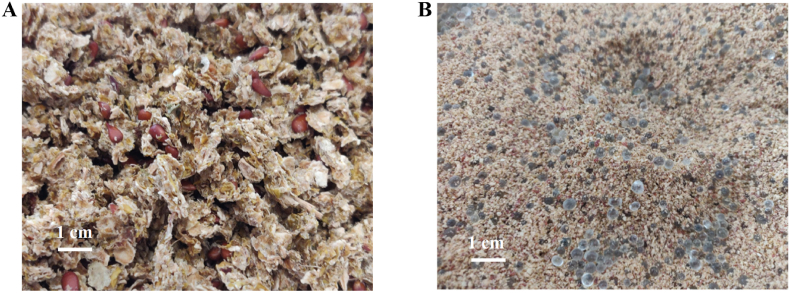
Dried pressing residue of Japanese quince before (A) and after milling mixed with glass beads for supercritical extraction (B).

The water content of the dried press residue (Fig. 1A) was 4.49 g/100 g, and the seed content of the dried residue was 42.4 ± 3.3 g/100 g of dried press residue, determined after manual separation of seeds from peel and pulp. Before extraction, the dried residue was ground with a cutting mill SM300 (Retsch, Germany) at 1500 rpm using a 1 mm sieve.

### 2.2 Supercritical CO_2_ extraction of the oil fraction

For the extraction of oil fraction, we packed 400 g of JQ pressing residue powder (particle size 0.3–0.5 mm) with 1578 g glass beads (Fig. 1B) into a 1.7 L extraction basket vessel. SC-CO_2_ extraction was conducted at 35 MPa and 50 °C, the CO_2_ flow rate was 200 g/min (SFE 2, Separex, France), and the separator temperature was 40 °C. These conditions were different from those used by (Górnaś et al., 2019) as our earlier results with modified conditions indicated higher oil extraction yields (unpublished data). The extraction time with pure CO_2_ was 120 min. With ethanol as a co-solvent (0.5 and 1% w/w of CO_2_), the extraction time was 90 min, followed by 15 min extraction with pure CO_2_. After extraction, ethanol was evaporated with a vacuum evaporator at 20 °C. All extractions were duplicated.

### 2.3 Total and free fatty acids analysis

Total fatty acids were extracted with a dichloromethane/methanol mix from JQ oil samples. The fatty acids (FA) were methylated using a mixture of methanol and acyl chloride. We conducted the analysis with an Agilent 6890A gas chromatograph equipped with split/splitless injector and a flame ionisation detector (FID). The studies used a quartz capillary column with liquid phase CP-Sil 88 (100 m × 0.25 mm, film thickness 0.20 μm). The results were expressed in peak area % of a specific FA of the total FA content.

### **2.4** Analysis of volatiles

JQ oil samples (10 μL) were diluted in 1 mL of hexane, and the solution was filtered with a 0.2 μm PTFE filter. GC/MS analyses were performed on an Agilent Technologies 7820A gas chromatograph coupled to an Agilent 5975C detector. A nonpolar HP-5MS column (30 m × 0.25 mm, 0.25 μm film thicknesses) with (5% phenyl)- methylpolysiloxane phase was used. The temperature program began at 40 °C (hold 1 min) and ramped to 250 °C at 7 K/min (hold 10 min). The inlet temperature was 250 °C, the split ratio 10:1, and the mass range from m/z 50–500. Identification of peaks was assigned against the NIST20 mass spectral library; spectral similarity was set to 80%.

### **2.5** Analysis of tocopherols and phytosterols

JQ oil samples (0.1 g) were dissolved in 5 mL of isopropanol. Tocopherols and phytosterols were quantitatively analysed on a Shimadzu Nexera X2 UHPLC (Shimadzu, Japan) coupled with MS detector LCMS 8040 (Shimadzu, Japan). The UHPLC system was equipped with a binary solvent delivery pump LC-30AD, an autosampler Sil-30AC and column oven CTO-20AC. A reverse phase column Ascentis Express (C30, 50 × 4.6 mm; Supelco) and pre-column (SecurityGuard ULTRA, C18; Phenomenex, USA) were used at 40 °C to separate the analytes. The mobile phase comprised isopropanol(A) and methanol (B); the flow rate was 0.5 mL/min and sample size was 1 μL. Separation was carried out for 15 min under the following conditions: isocratic 0–4 min, 99% B; gradient 4–5 min, 99–10% B; 5–10 min, 10% B, 10–12 min 10–99% B, and re-equilibration of the system with 99% B 3 min. MS data acquisitions were performed on LCMS 8040 (Shimadzu, Japan), with the APCI source operating in positive mode. The interface voltage was set to 4.5 kV. Nitrogen was used as the nebulizing gas (3 L/min) and drying gas (5 L/min). The heat block temperature was 350 °C, and the desolvation line (DL) temperature was 200 °C. Transitions for the analytes are given in Table S1.

### **2.6** Oxidation stability of oils

The oxidation stability of the JQ oil samples was first assessed by the Rancimat method. 3.5 g of oil samples in the Rancimat reaction vessel (Rancimat 892, Metrohm, Switzerland) were placed at 100 °C under a constant airflow rate of 10 L/h for accelerated oxidation. The oxidation results were expressed as induction time (h), showing the time required for the conductivity curve to reach the point of complete oxidation of oil samples. Oils’ peroxide values were determined following the ISO 3976:2006 method.

### 2.7 Ultrasound-assisted extraction of polyphenolic compounds

After SC extraction, the ultrasound-assisted extraction (UAE) of polyphenolic compounds from the defatted residue was performed using a UP 400St ultrasonic processor (⍉18 mm titanium horn; Hielscher GmbH, Germany). 10 g of dry powder was mixed with 250 mL ethanol–water solution (70:30, v/v). The extraction was carried out at 40% amplitude (80 W) for 15 min. The obtained extract was separated from the residual material by vacuum filtration.

### 2.8 Isolation of proteins

Protein isolates were extracted from defatted JQ residue recovered after SC-CO_2_ extraction of oil with or without extraction of polyphenolic compounds as described above to remove tannins. Protein extraction was performed following the method reported by Deng et al. (2019) with some modifications. The defatted JQ residue was dispersed in distilled water with a solid to liquid ratio of 1:20 (w/v), and the pH of the mixture was adjusted to pH 10 using 4 mol/L NaOH solution. The mixture was mixed for 2 h at room temperature and centrifuged at 4000×*g* for 30 min to recover the supernatant containing the extracted proteins. The supernatant was acidified to the isoelectric point at pH 4.2 using 1 mol/L HCl solution to precipitate proteins. The precipitated proteins were recovered by centrifugation at 4000×*g* for 30 min. The supernatant was discarded, the protein isolate was washed twice with water, and resuspended in distilled water, adjusting the pH to 7 using 4 mol/L NaOH solution. The protein isolate was freeze-dried and stored at 4 °C until further analyses.

### **2.9** Amino acid analysis

For amino acid analysis, LC-MS/MS system equipped with Agilent 1290 Infinity II quaternary pump, column thermostat, an autosampler and an Agilent 6460 Triple Quadrupole (QqQ) mass spectrometer (MS) with Agilent Jet Stream Technology electrospray ionisation source (ESI) was employed.

Protein isolates (ca 30 mg) were hydrolyzed in 10 mL of 0.1% phenol in 6 mol/L HCl in Ace pressure tubes (Sigma Aldrich, dimensions 10.2 cm × 25.4 mm) at 110 °C for 24 h. The solvent was replaced with 4.2 mol/L NaOH for tryptophan or 0.1% phenol and 3% thioglycolic acid in 6 mol/L HCl for cysteine. The sample was filtered through hydrophilic regenerated cellulose syringe filter Chromafil®Xtra (25 mm diameter, 0.20 μm pore diameter) and diluted 200 times with 30% methanol in 0.1 mol/L HCl, before derivatization with diethyl ethoxymethylenemalonate, according to the procedure by Maciel, Marengo, Rubiolo, Leito, & Herodes, K (2021), without the addition of the quenching reagent.

Chromatographic analysis was performed in a Zorbax Eclipse Plus C18 (3.0 × 100 mm, 1.8 μm) column and guard column (3.0 × 5 mm, 1.8 μm), maintained at 40 °C. The sample size was 2 μL. The mobile phase was composed of 0.1% aqueous formic acid (A) and acetonitrile (B), with the following gradient for B: 0–2 min, 10%; 2–27 min, 10–98%; 27–29 min, 98%; 29–31min, 98–10%; the total running and post-running time was 35 min. The eluent flow rate was 0.4 mL/min. The ESI and MS parameters were as follows: drying gas temperature 320 °C, drying gas flow 9 L/min, nebulizer gas pressure 45 psi (310264 Pa), sheath gas temperature 380 °C, sheath gas flow 12 L/min. The instrument was operated in positive ionisation mode: capillary voltage 3000 V and nozzle voltage 0 V. Dynamic multiple reaction monitoring (dMRM) was used with fragmentor 90 V and collision energy 8 V. Transitions for each amino acid are shown in Table S2.

### 2.10 Determination of protein functional properties

*Emulsification capacity* was determined by measuring the amount of oil (g) emulsified by protein dissolved in water until the emulsion turns from oil in water to water in oil emulsion. The turning point was detected by the increase in the emulsion's resistance monitored using an ohmmeter (Digital Multimeter, Fluke Corporation, USA). The protein solution (50 mL, 0.01 g/100 mL, pH7.00) was poured into the reaction cylinder and mixed using Ultra-Turrax homogenizer (IKA®-Werke GmbH & Co., Germany) at 13,000 rpm, and canola oil was gradually pumped to the solution (25 mL/min). When the emulsion's resistance increased over the detection limit of the ohmmeter, the oil pump was stopped and the amount of added oil determined. Emulsification capacity was calculated as follows:

(1) $$Emulsification\;capacity=\frac{Amount\;of\;oil\;added\;\left(g\right)}{Solution\;protein\;content\;\left(mg\right)}$$

*Emulsification activity and emulsion stability* were determined by measuring the ratio of stable emulsion in total oil/water dispersion. Briefly, 60 mL of protein dispersion (2 g/100 mL, pH7.00) was emulsified with 60 g of oil using Ultra-Turrax homogenizer at 13,000 rpm for 2 min. Then, 10 mL aliquots of the emulsion were transferred to 10 mL tubes and centrifuged at 2690 rpm for 5 min at room temperature. While for emulsion stability, tubes were incubated at 80 °C for 30 min, cooled down, and centrifuged. Emulsification activity and stability were calculated using the following equations:

(2) $$Emulsification\;activity\;\left(\%\right)=\frac{height\;of\;the\;emulsion}{height\;of\;total\;dispersion}\times 100$$

(3) $$Emulsion\;stability\;\left(\%\right)=\frac{height\;of\;the\;emulsion\;after\;heat\;treatment}{height\;of\;total\;dispersion\;after\;heat\;treatment}\times 100$$

*Foaming capacity and foam stability:* 100 mL of protein dispersion (1 g/100 mL, pH7.00) was whipped using Ultra-Turrax homogenizer at 16,000 rpm for 2 min. The volume of the foam was recorded at 0 min and after 30 min at room temperature. Foaming capacity and stability were calculated as follows:

(4) $$Foaming\;capacity\;\left(\%\right)=\frac{foam\;volume\;at\;0\;\min}{Initial\;dispersion\;volume}\times 100$$

(5) $$Foam\;stability\;\left(\%\right)=\frac{foam\;volume\;after\;30\;\min}{foam\;volume\;at\;0\;\min}\times 100$$

### 2.11 Determination of condensed tannins content

Condensed tannins were determined using 4-dimethylaminocinnamaldehyde (DMAC) reagent as described by Heil, Baumann, Andary, Linsenmair, & McKey, 2002 with modifications. Briefly, 10 mg of protein sample was extracted for 26 h in darkness with 1 mL of methanol. Further on, extracts were centrifuged at 13,000 rpm (Eppendorf MiniSpin). 2380 μL of methanol and 100 μL of the DMAC reagent were added to 20 μL of catechin standard (50–500 mg/L) or sample. After reaction at room temperature for 15 min in darkness, absorbance was measured at 640 nm using a UV–Vis spectrophotometer.

### 2.12 Analysis of polyphenols and amygdalin

Polyphenols and amygdalin were analysed on a Shimadzu Nexera X2 UHPLC coupled with mass spectrometer LCMS 8040 (Shimadzu, Japan) as described in Ben-Othman et al. (2021) with slight modifications. A reverse phase column ACE Excel 3 (C18, PFP, 100 × 2.1 mm; from ACE® Advanced Chromatography Technologies Ltd., Scotland) and pre-column (SecurityGuard ULTRA, C18; from Phenomenex, USA) were used at 40 °C for the separation of individual compounds. The flow rate of the mobile phase was 0.4 mL/min; the sample size was 1 μL. The mobile phase consisted of 1% formic acid in Milli-Q water (A) and 1% formic acid in methanol (B). Separation was carried out for 38 min under the following conditions: gradient 0–10 min, 10–25% B; 10–15 min, 25–35% B; 15–27 min, 35%–80% 27–30min 80-95 29–34 min isocratic 95% B, and re-equilibration of the system with 10% B 5 min.

Phenolic compounds were identified by comparing the retention times, UV spectra, and parent/daughter ion masses with those of the standard compounds described in Ben-Othman et al. (2021).

### 2.13 Statistical analysis

All extractions were duplicated, and further analyses were performed in triplicates. Results are expressed as mean ± standard deviation. The statistical analysis was conducted using Prism 5 (GraphPad Software, San Diego, CA, USA). Data were analysed by one-way analysis of variance (ANOVA), followed by Tukey's test.

## 3 Results and discussion

### 3.1 Oil fraction

#### 3.1.1 The extraction yield

SC-CO_2_ extraction represents an attractive alternative to conventional oil extraction methods using hazardous organic solvents, and this technique has been widely used for oil extraction from oleaginous nuts and seeds (Sahena et al., 2009). Górnaś et al. (2019) showed that SC-CO_2_ extraction of oil from quince seeds gave a higher yield than cold-pressing extraction. However, SC-CO_2_ extraction yield was lower than that of Soxhlet and UAE using n-hexane as a solvent. Therefore, SC-CO_2_ was selected for a safe (green) and high-yield extraction of oil from JQ pressing residue in the present work. Ethanol was used as a polar co-solvent to evaluate its effect on extracting polar bioactive compounds and oil quality.

**Fig. 2:**
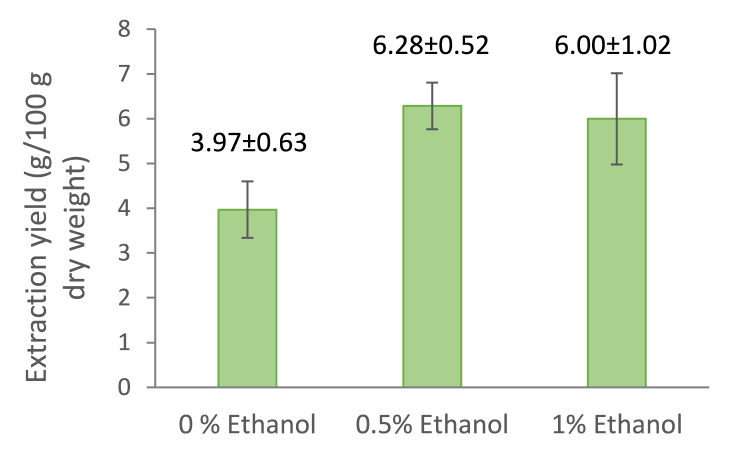
JQ pressing residue oil extraction yield after SC-CO_2_ extraction using ethanol as a co-solvent. The results are mean values ± standard deviation, n = 3.

The extraction yield of oil was 3.97 ± 0.63 g/100 g of the initial dry weight of the JQ residue with pure CO_2_ (Fig. 2). The obtained yield is in a good correlation with the oil extraction yield of 9.4 g/100 g from cv. ‘Rasa’ seeds reported by Górnaś et al. (2019) since the seed content of JQ press residue used in the present study was 42%. The oil yield increased to 6.28 ± 0.52 and 6.00 ± 1.02 g/100 g when 0.5 and 1% ethanol was added as a co-solvent, respectively. Using a polar co-solvent increases the polarity of the supercritical fluid and enhances the co-extraction of polar compounds such as polyphenols, phospholipids, and pigments, which can also ameliorate the quality of the obtained oil (de O. Silva, Ranquine, Monteiro, & Torres, 2019).

#### 3.1.2 Fatty acid composition

Twenty-two different fatty acids (FAs) were identified by GC/MS analysis in oil samples extracted with or without ethanol as a co-solvent and there was no statistically significant difference in FA contents between different extracts (Tukey's test P < 0.05) (Table 1).Linoleic acid constituted 50%, oleic acid 32%, and palmitic acid 10.5% of the total content of FAs, which is similar to the FA composition, reported in cold-pressed oil from JQ seeds (Górnaś et al., 2013). The referred FAs have several health-beneficial effects. The main n-6 FA - linoleic acid, is an essential polyunsaturated FA with a positive impact on cardiovascular health and cognitive functions (Arbex et al., 2015), while the monounsaturated oleic acid, which is also the main component of olive oil (70–80%) has a hypotensive effect (Teres et al., 2008). The ratio of saturated and unsaturated FAs in JQ extracts was about seven.

**Table 1:** The fatty acid composition of Japanese quince oil extracts.

Fatty acid		Control	0.5% Ethanol	1% Ethanol
***Saturated***				
Caprylic acid	C8:0	0.43 ± 0.01	0.43 ± 0.01	0.42 ± 0.01
Capric acid	C10:0	0.11 ± 0.004	0.11 ± 0.01	0.10 ± 0.01
Lauric acid	C12:0	0.16 ± 0.01	0.17 ± 0.001	0.18 ± 0.004
Myristic acid	C14:0	0.20 ± 0.02	0.20 ± 0.04	0.21 ± 0.02
Pentadecylic acid	C15:0	0.05 ± 0.004	0.05 ± 0.01	0.05 ± 0.01
Palmitic acid	C16:0	10.40 ± 0.08	10.53 ± 0.27	10.48 ± 0.14
Stearic acid	C18:0	1.18 ± 0.05	1.29 ± 0.18	1.31 ± 0.17
Arachidic acid	C20:0	0.63 ± 0.01	0.63 ± 0.01	0.63 ± 0.01
Behenic acid	C22:0	0.20 ± 0.02	0.22 ± 0.005	0.22 ± 0.02
Lignoceric acid	C24:0	0.14 ± 0.01	0.15 ± 0.02	0.14 ± 0.01
Total		13.49 ± 0.19	13.78 ± 0.59	13.74 ± 0.35
***Monounsaturated***				
*cis*-7-Hexadecenoic acid	C16:1c7	0.06 ± 0.02	0.06 ± 0.01	0.07 ± 0.004
Palmitoleic acid	C16:1c9	0.20 ± 0.05	0.23 ± 0.01	0.24 ± 0.01
*Cis*-10-heptadecenoic acid	C17:1c9	0.06 ± 0.002	0.05 ± 0.003	0.06 ± 0.005
Elaidic acid	C18:1t	0.06 ± 0.01	0.04 ± 0.01	0.06 ± 0.03
Oleic acid	C18:1c9	32.00 ± 0.04	31.99 ± 0.06	32.08 ± 0.12
Cis-Vaccenic acid	C18:1c11	1.52 ± 0.02	1.47 ± 0.12	1.46 ± 0.10
*Cis*-13-Octadecenoic acid	C18:1c13	0.14 ± 0.02	0.15 ± 0.01	0.13 ± 0.02
Gondoic acid	C20:1c	0.65 ± 0.02	0.67 ± 0.04	0.66 ± 0.04
Total		34.69 ± 0.06	34.66 ± 0.06	34.76 ± 0.13
***Polyunsaturated***				
Linoleic acid	C18:2n6	50.03 ± 0.22	49.70 ± 0.55	49.70 ± 0.32
α-linolenic acid	C18:3n3	1.47 ± 0.04	1.53 ± 0.03	1.52 ± 0.04
eicosadienoic acid	C20:2n6	0.15 ± 0.03	0.14 ± 0.01	0.14 ± 0.01
Dihomo-γ-linoleic acid	C20:3n6	0.05 ± 0.01	0.05 ± 0.02	0.04 ± 0.01
Total		51.70 ± 0.18	51.42 ± 0.58	51.40 ± 0.34

All values are means ± standard deviation, *n* = 3. No significant statistical difference between different extracts.

The use of ethanol as a co-solvent did not affect the content and composition of FAs in oil extracts. Górnaś et al. (2019) have also reported that although the use of different extraction methods has a significant impact on JQ seed oil yield, the FA composition of the obtained oil is not affected by choice of the extraction method, which is in accordance with our results.

Interestingly, the composition of minor FAs of oil extracted from JQ press residue slightly differs from oil obtained from JQ seeds. The FA analyses indicated the presence of very low amounts of short-chain saturated FAs like C8:0, C10:0, and C12:0, which have not been detected in cold-pressed JQ seed oil (Górnaś et al., 2019). In addition, JQ press residue oil contains up to 1.53 g/100 g of α-linolenic acid (α-LA), which is almost 3-fold higher than α-LA content previously reported in JQ seed oil (0.56 g/100 g) (Górnaś et al., 2019). α-LA is an essential omega-3 FA metabolized into docosahexaenoic acid (DHA), which has a crucial role in maintaining cardiovascular health and anti-inflammatory and neuroprotective properties (Barceló-Coblijn & Murphy, 2009; Pan et al., 2012). The increased α-LA content in JQ oil can be explained by the fact that in this study, oil was extracted from fruit press residue, which also contained skin and pulp beside seeds, while previous studies used only seeds for oil extraction. The ratio of omega-6 to omega-3 FAs in JQ press residue oil is about three times lower (34:1) compared to cold-pressed JQ seed oil (93.5:1).

In addition, JQ press residue oil contains 1.46–1.52 g/100 g of *cis*-vaccenic acid, which has not been detected in JQ seed oil. We suggest that this FA was also extracted from the pulp portion of JQ press residue since previous studies have reported the presence of *cis*-vaccenic acid in fruit pulp such as sea buckthorn (*Hippophae rhamnoides* L.) and persimmon (*Diospyros kaki*) (Pchelkin, Kuznetsova, Tsydendambaev, & Vereshchagin, 2006).

#### 3.1.3 The content of polyphenols

The use of ethanol as a co-solvent did not considerably affect the concentrations of polyphenolic compounds in the oil extracts. The only polyphenolic compound detected was quinic acid. The concentration of quinic acid in oils from ethanol-free extraction was 292 ± 75 μg/mL, and it increased along with the increase of ethanol concentration, being 739 ± 168 μg/mL and 1205 ± 194 μg/mL for 0.5% and 1.0% ethanol, respectively. It has been proposed that quinic acid may possess antimicrobial and antiviral properties (Benali et al., 2022), which may increase the resistance to microbial growth and favour a better microbiological storage stability of the oil. The concentrations of all other polyphenolic compounds in oil extracts were minute.

#### 3.1.4 Oxidative stability

As JQ is rich in unsaturated fatty acids, oxidative stability is among the major indicators assessing JQ oil quality. The stability of oil depends mainly on FA composition but also on the content of antioxidants and other compounds extracted along with oils (Symoniuk, Ratusz, Ostrowska-Ligeza, & Krygier, 2018).

First, we evaluated the oxidation stability with the widely used Rancimat method following the change of water conductivity in a measuring vessel due to the inflow of volatile compounds formed during the accelerated decomposition of oils at higher temperatures. The JQ waste oils had very low induction times, especially for oils extracted using ethanol as a co-solvent. With 0.5% ethanol added, the induction time of oil was 0.24 h, and with 1% ethanol added, it was 0.52 h. The extract obtained with CO_2_ only showed an induction time several times higher: 2.62 h. The latter value is comparable with the induction times of other edible plant oils, commonly being over 2 h (at 120 °C) (Maszewska et al., 2018). Although conductivity increase and induction time decrease can be caused by certain fatty acids of low molecular weight volatilising at 100 °C, such FAs were not identified in JQ oils. The composition of volatile compounds in different extracts was profiled with GC/MS (Table 2).

**Table 2:** Volatile compounds detected in Japanese quince residue oil extract.

RT (min)	Name	Control		0.5% ethanol		1.0% ethanol	
7.99	Benzaldehyde	+	+	+	+	+	+
9.66	Benzyl alcohol		+	+	+	+	
9.69	1,2-Ethanediol, 1,2-diphenyl-, (R*,R*)-(.+/−.)-	+					
11.16	Nonanal	+	+	+	+	+	
12.52	Octanoic acid	+	+	+	+	+	+
12.66	Benzoic acid, ethyl ester	+	+			+	
13.22	Estragole		+				
13.81	Benzaldehyde diethylacetal			+	+	+	+
13.90	2-Methyl-1-phenylbut-3-en-1-ol				+	+	
13.92	1,2-Ethanediol, 1-phenyl-			+			
16.20	3-Decenoic acid, (E)-	+	+	+	+	+	
16.84	2-Propenal, 3-phenyl-	+	+	+	+	+	+
20.54	Hexadecane					+	
25.99	n-Hexadecanoic acid	+	+	+	+	+	+
26.46	Hexadecanoic acid, ethyl ester	+	+	+	+	+	+
28.35	9,12-Octadecadienoic acid (Z,Z)-	+	+	+	+	+	+
28.41	cis-Vaccenic acid	+	+	+	+	+	+
28.70	Linoleic acid ethyl ester	+	+	+	+	+	+
28.76	(E)-9-Octadecenoic acid ethyl ester	+	+	+	+	+	+
28.91	Hexadecanamide				+		+
31.14	9-Octadecenamide, (Z)-	+	+	+	+	+	+

(+) indicates the presence of the volatile compound in the analysed sample.

The major difference in the composition of volatile compounds in different extracts was related to the concentration of benzaldehyde and its diethylacetal derivative (Table 2). Those obtained with pure CO_2_ contained almost three times more benzaldehyde than the extracts obtained with adding ethanol, and the concentration of benzaldehyde diethylacetal in these extracts was negligible (Fig. S1). However, if ethanol was used as a co-solvent during supercritical CO_2_ extraction, benzaldehyde diethylacetal was found in all extracts of quince oil, probably resulting from the acetalization of benzaldehyde with ethanol during the extraction or evaporation processes. Benzaldehyde diethylacetal readily undergoes hydrolysis in water, producing benzaldehyde and ethanol. The latter is evaporated at temperatures below 100 °C (boiling point at 78.4 °C), while both benzaldehyde (bp 178.1 °C) and benzaldehyde diethylacetal (bp 239.9 °C) stay in liquid phase, so causing the increase of conductivity and the decrease of induction time.

**Fig. 3:**
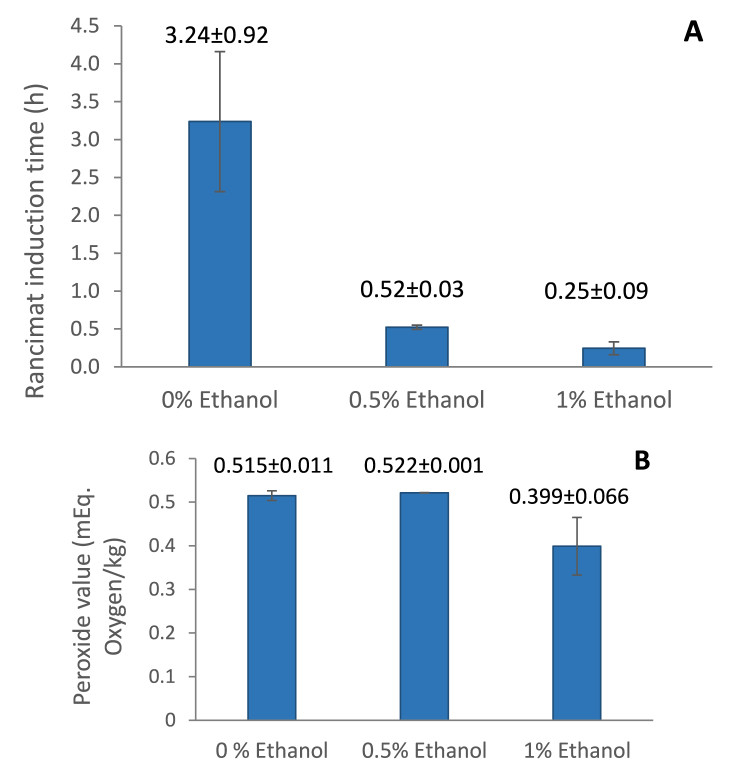
Oxidative stability of JQ press residue oil. (A) Rancimat® induction time, (B) Peroxide value. The results are mean values ± standard deviation, n = 3.

To assess of the oxidation stability, we also determined the peroxide value of the JQ oil samples. The content of primary oxidation products was similar in all extracts obtained with pure CO_2_ or ethanol added, indicating that the use of ethanol as a co-solvent does not have an essential impact on the stability of JQ oil (Fig. 3).

#### 3.1.5 The content of tocopherols and phytosterols

The content of tocopherols in JQ oils was relatively high, ranging from 71.4 to 96.2 mg/100 g oil (Table 3). The major tocopherol in all JQ oil extracts, constituting ∼95% of all tocopherols, was α-tocopherol, the most common in nature tocopherol with the highest biological activity (Reboul, 2017). The contents of β- and γ-tocopherols were considerably lower but still detectable, while the content of δ-tocopherol was under the limit of quantification (10 pg/mL). All four forms of tocopherol and tocotrienols, are known as different forms of vitamin E, known for their antioxidant function to terminate lipid self-oxidation. However, as other vitamin E forms are interconverted to α-tocopherol in humans, the dietary requirements are currently limited to α-tocopherol (Bruno & Mah, 2014).

**Table 3:** The content of tocopherols and phytosterols in Japanese quince residue oil.

	Control (no ethanol), mg/100 g oil	0.5% ethanol, mg/100 g oil	1% ethanol, mg/100 g oil
***Tocopherols***			
α-tocopherol	92.2 ± 10.5 ^a^	67.8 ± 3.4 ^b^	77.2 ± 11.7 ^ab^
β- and - γ-tocopherols combined	3.1 ± 0.5 ^a^	2.8 ± 0.3 ^a^	3.3 ± 0.5 ^a^
***Phytosterols***			
β-sitosterol	1422.8 ± 120.0 ^a^	1268.2 ± 22.7 ^a^	1490.9 ± 261.8 ^a^
Stigmasterol	23.2 ± 3.1 ^a^	19.4 ± 0.9 ^a^	23.2 ± 4.4 ^a^
Campesterol	81.6 ± 8.0 ^a^	71.1 ± 1.3 ^a^	85.0 ± 14.8 ^a^

All values are means ± standard deviation, *n* = 3; mean values within the same row with different letters are significantly different at *p* < 0.05.

JQ oil fractions also contained a high amount of phytosterols, mainly β-sitosterol with 1268.2–1490.9 mg/100 g oil (Table 3). Phytosterols are plant analogues of cholesterol that are essential in reducing blood cholesterol levels and preventing cardiovascular disease (Feng et al., 2020). Phytosterols have also been reported to reduce the risk of different cancers (Blanco-Vaca & Julve, 2019). Edible oils and nuts are the main dietary source of phytosterols. Bai, Ma, and Chen (2021) reported the phytosterol content of several edible oils; wheat germ oil had the highest phytosterol content (4240 mg/100 g oil), while common edible oils like olive, sunflower, and rapeseed oils contained 283 mg, 451 mg, and 887 mg per 100 g respectively. Thus, JQ oil tocopherols and phytosterols contents make it an interesting functional food ingredient.

### 3.2 Protein fraction

#### 3.2.1 Extraction yield

Protein isolates were extracted from the defatted JQ residue of SC-CO_2_ extraction using two approaches: 1) the residue was directly used for protein extraction, and 2) tannins were removed by UAE using 70% ethanol before the extraction of proteins. The yield and composition of protein isolates from defatted JQ residue after SC-CO_2_ extraction with or without ethanol as co-solvent were similar; thus we summarized all results in Table 5.

**Table 4:** Protein isolate extraction yield and tannins content.

	Extraction method 1	Extraction method 2
Initial protein content^a^ (g/100 g)	11.42 ± 0.61	
Protein isolate yield (g/100 g)	6.04 ± 0.99	2.58 ± 0.27
Tannins content (mg C.Eq./g of PI)^b^	4.45 ± 1.92	0.12 ± 0.11

All values are means ± standard deviation, *n* = 3.

a Protein content determined by Kjeldhal method.

b Tannins content is expressed as mg catechin equivalent per g of protein isolate.

**Table 5:** Amino acid composition of protein isolate from Japanese quince residue.

	Amino acid content (mg/100 mg protein)	Essential amino acid requirements for adult (mg/100 mg protein) according to WHO/FAO
*Essential amino acids (EAA)*		
Histidine	2.16 ± 0.11	1.5
Threonine	2.83 ± 0.10	2.3
Tyrosine	3.33 ± 0.11	3.8 (Tyr + Phe)
Methionine	0.98 ± 0.09	1.6
Valine	3.10 ± 0.07	3.9
Tryptophan	0.93 ± 0.07	0.6
Phenylalanine	4.74 ± 0.12	3.8 (Tyr + Phe)
Isoleucine	2.54 ± 0.04	3.0
Lysine	3.12 ± 0.13	4.5
Leucine	7.32 ± 0.34	5.9
*Non-essential amino acids (NEAA)*		
Arginine	10.02 ± 0.11	
Serine	4.45 ± 0.23	
Aspartic acid	9.77 ± 0.17	
Glutamic acid	28.98 ± 1.26	
Glycine	6.25 ± 0.12	
Alfa-Alanine	4.49 ± 0.16	
Proline	2.44 ± 0.05	
Cysteine	2.55 ± 0.18	

All values are means ± standard deviation, *n* = 3.

It is not possible to determine asparagine and glutamine by protein digestion. They are converted into aspartic and glutamic acid, respectively.

The extraction of the defatted JQ residue with 70% ethanol removed the majority of tannins co-extracted with protein fraction and the catechin equivalent decreased from 4.45 to 0.12 mg C.Eq./gPI (Table 4). The individual phenolic compounds analysis (Table S3) showed that protein isolates mainly contained condensed tannin procyanidin B2 (150.5 μg/g) and monomeric tannins like catechin (29.7 μg/g) and epicatechin (89.2 μg/g). Tannins form complexes with proteins reducing protein solubility and digestibility and leading to low amino acid bioavailability (Ram, Narwal, Gupta, Pandey, & Singh, 2020). However, the extraction yield of the protein isolates obtained after ethanol extraction was significantly lower, decreasing from 6.04 ± 0.99 to 2.58 ± 0.27 g/100 g, which indicates that part of the protein fraction was also extracted by ethanol.

#### 3.2.2 Amino acid composition

The amino acid (AA) composition of JQ protein isolate is presented in Table 5. Glutamic acid (28.98 mg/100 mg), arginine (10.02 mg/100 mg), and aspartic acid (9.77 mg/100 mg) were the major AAs in JQ protein isolates which is similar to AA composition of protein isolate of Chinese quince seeds (*Chaenomeles speciosa*) (Deng et al., 2019). Czubinski and co-authors (2021) analysed the AA composition of proteins extracted from JQ seeds at different pH values (pH 2–9), which contained a higher content of essential AAs (histidine, lysine, leucine, and phenylalanine) and lower amount of non-essential AAs (glutamic and aspartic acids) compared to our results. The difference in the extraction conditions can explain this. Amino acid analysis also revealed that JQ protein isolate contains most of the essential amino acids, except for methionine, valine, and lysine, in sufficient amounts to fulfil the requirements for adult diets according to World Health Organization and Food and Agriculture Organization recommendations (World Health Organization, 2002) (Table 5). This high content of essential amino acids in the JQ protein fraction also indicates that JQ fruit wine residue can be a good protein source.

#### 3.2.3 Functional properties

The emulsifying and foaming functional properties of JQ protein isolate are presented in Table 6. JQ protein isolate showed excellent emulsifying properties; its emulsifying capacity was three-fold higher than that of hemp seed protein concentrate. Emulsifying activity and emulsion stability of JQ protein isolate were also higher compared to hemp protein. Deng et al. (2019) have also reported that Chinese quince seed protein isolate has higher emulsifying activity than soy protein isolate, a widely used emulsifier in food industry. This suggests that proteins of *Chaenomeles* genus fruits’ seeds have interesting emulsifying properties and high potential as an emulsifying agent in food industry. Thanks to their amphiphilic nature, proteins behave as emulsifiers by adsorbing at the oil/water interface surrounding oil droplets and stabilizing the emulsion. Protein structure, molecular weight, and amino acid composition influence its behaviour at the oil/water interface and, thus, its emulsifying properties (Kim, Wang, & Selomulya, 2020). Therefore, further studies are still needed to analyse the structure of JQ proteins and understand their functional properties.

**Table 6:** Functional properties of JQ protein isolate and hemp seed protein concentrate.

Functional Property	JQ protein isolate	Hemp seed protein concentrate
Emulsifying capacity (g oil/mg)	71.97 ± 5.31	21.47 ± 1.12
Emulsifying activity (%)	82.8 ± 1.7	55.0 ± 0.6
Emulsion stability (%)^a^	74.3 ± 0.5	60.6 ± 1.0
Foaming capacity (% over-run)	52.0 ± 2.0	113.3 ± 14.4
Foam stability (%)^b^	80.6 ± 0.9	59.0 ± 0.7

All values are means ± standard deviation, *n* = 3.

a Emulsion was incubated 30 min at 80 °C.

b Foam stability was measured after 30 min at room temperature.

Besides, JQ protein isolate exhibited a relatively low foaming capacity but good foam stability compared to hemp protein concentrate. Foaming properties depend on protein solubility, molecular flexibility, surface charge, and hydrophobicity (Amagliani, Silva, Saffon, & Dombrowski, 2021).

### 3.3 Safety of JQ residue extracts

In addition to the high content of health-beneficial compounds, JQ seeds contain amygdalin, a cyanogenic glycoside, including a nitrile group metabolized into cyanide anion in the human body. Amygdalin is mainly found in seeds of *Rosaceae* species. The analyses of different fractions recovered from JQ press residue revealed the absence of amygdalin in all oil extracts obtained with or without ethanol as a co-solvent (Table 7). Amygdalin was extracted along with polyphenol extracts, its content being up to 117.7 ± 0.6 μg/mL, corresponding to 3080.3 ± 15.8 μg/g DW of defatted JQ residue. While protein isolates recovered with or without removing of tannins were amygdalin free.

**Table 7:** The content of amygdalin in different extracts from JQ press residue.

Extract		Amygdalin content (μg/mL)
Oil fraction	SC-CO_2_ extract	N.D.^a^
	SC-CO_2_ + 0.5% EtOH extract	N.D.
	SC-CO_2_ + 1% EtOH extract	N.D.
Polyphenols fraction	70% EtOH extract	117.7 ± 0.6
Protein fraction	Alkaline extraction from SC-CO_2_ residue	N.D.
	Alkaline extraction after EtOH extraction of polyphenols from SC-CO_2_ residue	N.D.

All values are means ± standard deviation, *n* = 3.

a N.D. means not detected, detection limit is 10 pg/mL.

## 4 Conclusions

The present work successfully recovered oil and protein fractions from JQ press residue using SC-CO_2_ and alkaline aqueous extractions, respectively. SC-CO_2_ extraction results showed that using of ethanol as a co-solvent to increase solvent polarity led to higher oil extraction yields. However, adding a co-solvent did not increase the oil oxidative stability as expected, as the Rancimat assay showed shorter induction times potentially related to the presence of different volatile compounds in oil extracted with ethanol as a co-solvent. The oil obtained from JQ residue is rich in PUFAs, mainly ω-6 linoleic acid. It also contains high concentrations of α-tocopherol and phytosterols, mainly β-sitosterol, well-known for their health-promoting properties and potential skin care bioactives.

The remaining defatted residue was further utilized to recover proteins after tannins were removed with 70% ethanol ultrasound-assisted extraction. The protein extraction yield was 2.58 ± 0.27 g/100 g, only 22.6% of the JQ residue's initial protein content. JQ protein isolate has a well-balanced amino acid composition containing all essential amino acids and exhibited interesting functional properties as an emulsifying agent with potential use as a food ingredient.

In conclusion, JQ fruit wine by-product containing up to 42 g/100 g seeds of its dry weight has a high added value as a source of oil and protein fractions that can be used as food or cosmetic ingredients.

## Funding

This research was funded by the European Union's Horizon 2020 Research and Innovation Program project VALORTECH under grant agreement No. 810630 and the Mobilitas Pluss ERA Chair support (no. MOBEC006). European Regional Development Fund is acknowledged for providing research collaboration.

## CRediT authorship contribution statement

**Sana Ben-Othman:** Methodology, Investigation, Data curation, Validation, Writing – original draft, Writing – review & editing, Visualization. **Uko Bleive:** Investigation, Formal analysis, Methodology. **Hedi Kaldmäe:** Investigation, Formal analysis, Writing – original draft. **Alar Aluvee:** Investigation, Formal analysis. **Reelika Rätsep:** Investigation, Formal analysis, Writing – original draft. **Kadri Karp:** Resources, Writing – review & editing. **Larissa Silva Maciel:** Investigation, Formal analysis, Data curation, Writing – original draft. **Koit Herodes:** Supervision, Methodology, Data curation. **Toonika Rinken:** Conceptualization, Methodology, Writing – original draft, Writing – review & editing, Visualization, Project administration.

## Declaration of competing interest

The authors declare no conflict of interest in this study.

## Data Availability

Data will be made available on request.
