# Supplementary material for: Phytochemical characterization of oil and protein fractions isolated from Japanese quince (Chaenomeles japonica) wine by-product
Source: Lebensm Wiss Technol. 2023 Mar 15;178:114632. doi: 10.1016/j.lwt.2023.114632 (PMC10028728; doi:10.1016/j.lwt.2023.114632)

**Table S1.** MRM transitions of tocopherols and phytosterols and their respective retention times

| Compound | Parent ion [M+H]+ | Quantifier product ion m/z | Ret Time (min) |
| --- | --- | --- | --- |
| b-Sitosterol | 397.4 | 81.15 | 3.99 |
| stigmasterol | 395.4 | 83.15 | 3.6 |
| campesterol | 383.4 | 80.95 | 3.67 |
| β-tocopherol | 417.4 | 151.0 | 2.54 |
| α-tocopherol | 430.4 | 165.1 | 2.85 |
| γ-tocopherol | 417.5 | 123.1 | 2.54 |

**Table S2**. Dynamic MRM transitions of each amino acid together with their respective retention time.

| **Amino acid** | **Precursor ion** | **Product ion** | **Retention time**  **(min)** |
| --- | --- | --- | --- |
| Arginine | 345 | 299 | 6.45 |
| Aspartic acid | 304 | 258 | 8.15 |
| Cysteine | 462 | 416 | 14.08 |
| Glutamic acid | 318 | 272 | 8.68 |
| Glycine | 246 | 200 | 8.88 |
| Histidine | 326 | 280 | 4.84 |
| Isoleucine | 302 | 256 | 14.40 |
| Leucine | 302 | 256 | 14.60 |
| Lysine | 441 | 395 | 14.83 |
| Methionine | 320 | 274 | 12.82 |
| Phenylalanine | 336 | 290 | 14.38 |
| Proline | 286 | 240 | 10.53 |
| Serine | 276 | 230 | 7.69 |
| Threonine | 290 | 244 | 8.83 |
| Tryptophan | 375 | 329 | 13.77 |
| Tyrosine | 352 | 306 | 11.17 |
| Valine | 288 | 242 | 12.98 |

**Table S3**. The content of individual phenolic compounds in JQ residue extracts

| **Sample** | **Quinic acid** | **Procyanidin B1** | **Rutin** | **Kaempferol-3-gluc** | **Quercetin** | **Catechin** | **Chlorogenic acid** | **Procyanidin B2** | **Procyanidin C3** | **Epicatechin** |
| --- | --- | --- | --- | --- | --- | --- | --- | --- | --- | --- |
| SC-CO_2_ only extract | 292 ± 75 µg/mL | N.D.* | N.D. | N.D. | N.D. | N.D. | N.D. | N.D. | N.D. | N.D. |
| SC-CO_2_ + 0.5% EtOH extract | 739 ± 168 µg/mL | N.D. | N.D. | N.D. | N.D. | N.D. | N.D. | N.D. | N.D. | N.D. |
| SC-CO_2_ + 1% EtOH extract | 1205.5 ± 194 µg/mL | N.D. | N.D. | N.D. | N.D. | N.D. | N.D. | N.D. | N.D. | N.D. |
| 70% EtOH extracts | A.D.L. (High)** | 0.83 ±0.22 µg/mL | 0.35 ± 0.03 µg/mL | 0.098 ± 0.01 µg/mL | 0.65 ± 0.09 µg/mL | 0.73 ± 0.06 µg/mL | 11.57 ±1.41 µg/mL | 106.77 ±3.24 µg/mL | 46.20 ±6.22 µg/mL | 55.50 ±3.12 µg/mL |
| Protein isolate 1 | 283.5 ± 134 µg/g | N.D. | 2.2 ± 1.5 µg/g | 1.2 ±0.7 µg/g | N.D. | 29.7 ± 16.0 µg/g | N.D. | 150.5 ±136.2  µg/g | 16.6 ± 0.01 µg/g | 89.2 ± 14.7 µg/g |
| Protein isolate 2 (after tannins removal) | 76.8 ± 5.2 µg/g | N.D. | 2.2 ± 1.6 µg/g | 0.5 ±0.7 µg/g | N.D. | N.D. | N.D. | 4.6 ± 0.01 µg/g | N.D. | N.D. |

All values are means ± standard deviation, *n* = 3.

* N.D. means not detected,

** A.D.L. means above detection limit

**Figure S1**. Profiles of volatile compounds of JQ oil. Peak detection threshold was set to minimum height of 1000 counts.

A: SC-CO_2_ only extract


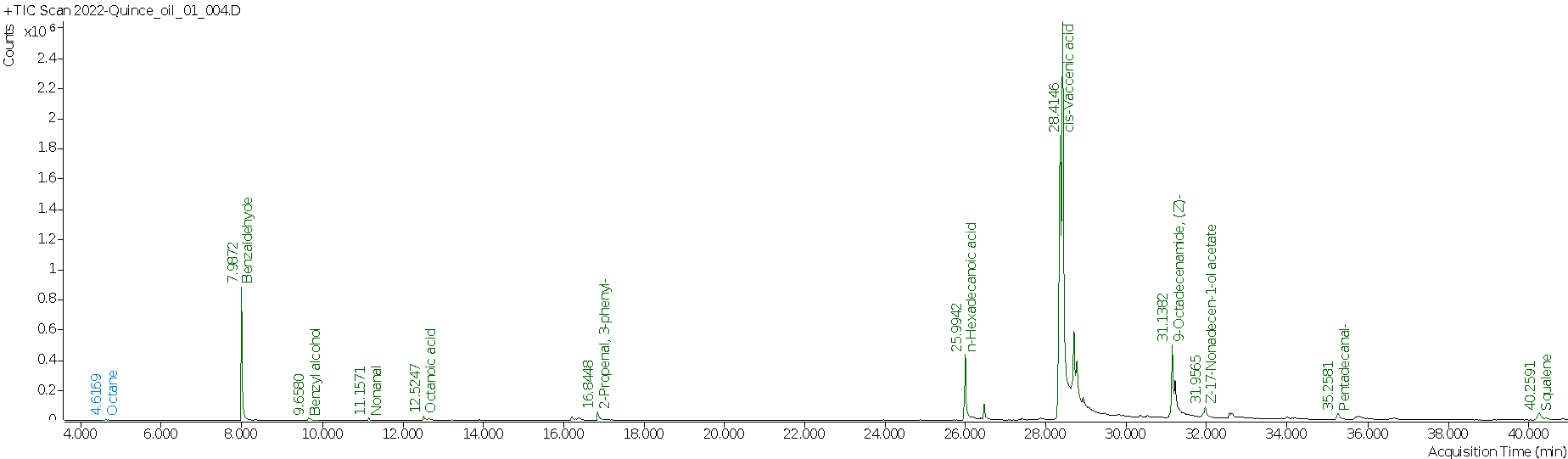


B: SC-CO_2_ + 0.5% EtOH extract


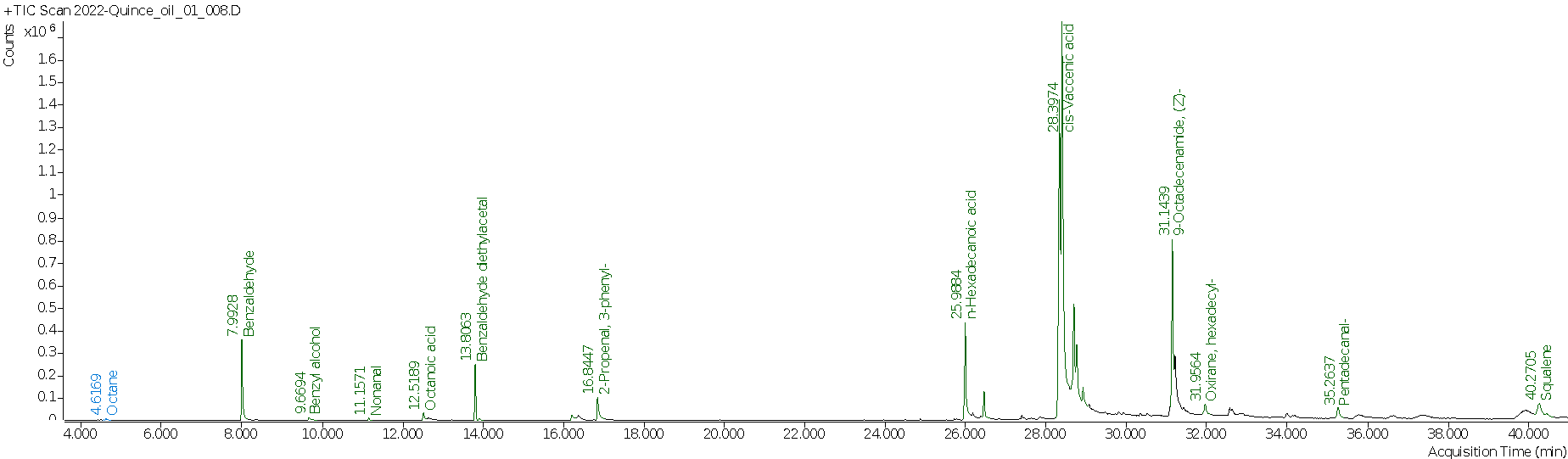


C: SC-CO_2_ + 1% EtOH extract


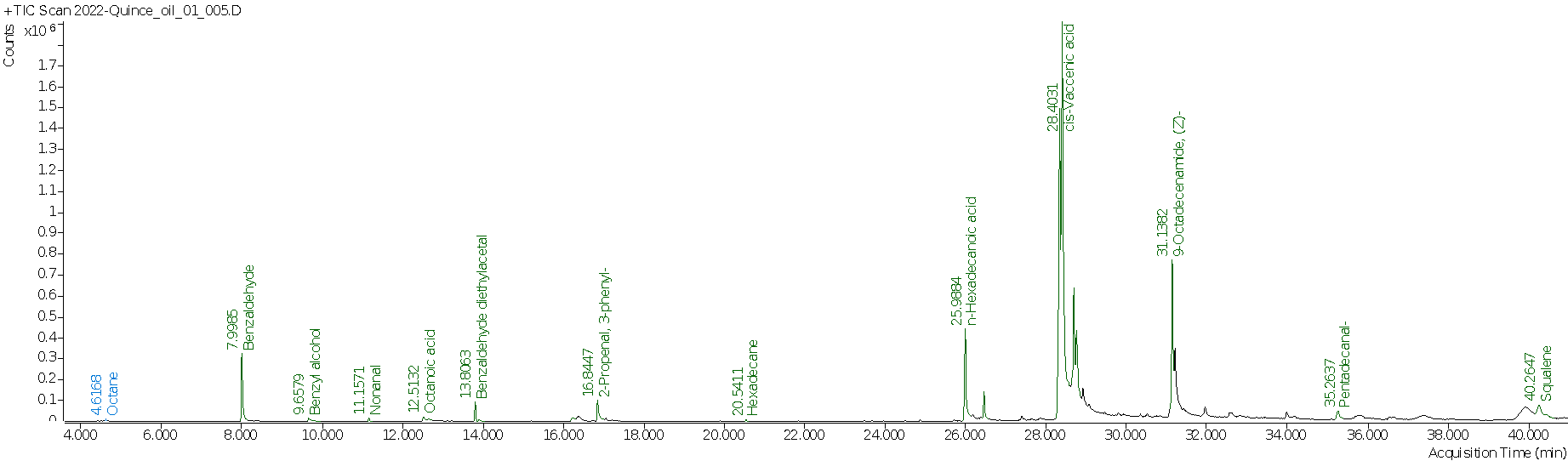

Supplement: Multimedia component 1 [file mmc1.docx]
